# Crystal structure of (1*S*,3*R*,8*R*,9*R*,10*S*)-2,4,6-tris­(2,2-di­chloro-3,7,7,10-tetra­methyl­tri­cyclo­[6.4.0.0^1,3^]dodec-9-yl)cyclo­triboroxane

**DOI:** 10.1107/S2056989015013390

**Published:** 2015-07-17

**Authors:** Ahmed Benharref, Lahcen El Ammari, Mohamed Saadi, Noureddine Mazoir, Moha Berraho

**Affiliations:** a Laboratoire de Chimie des Substances Naturelles, "Unité Associé au CNRST (URAC16)", Faculté des Sciences Semlalia, BP 2390 Bd My Abdellah, 40000 Marrakech, Morocco; b Laboratoire de Chimie du Solide, Appliquée, Faculté des Sciences, Université Mohammed V-Agdal, Avenue Ibn Battouta, BP 1014 Rabat, Morocco

**Keywords:** crystal structure, oil of the Atlas cedar (*Cedrus Atlantica*), cyclo­triboroxane

## Abstract

The title compound, C_48_H_75_B_3_Cl_6_O_3_, was synthesized in two steps from β-himachalene (3,5,5,9-tetra­methyl-2,4a,5,6,7,8-hexa­hydro-1*H*-benzo­cyclo­hept­ene), which was isolated from the essential oil of the Atlas cedar (*Cedrus Atlantica*). The mol­ecule consists of an almost planar cyclo­triboroxane ring [maximum deviation = 0.036 (2) Å] linked to three identical fused ring systems with different conformations. Each of the three attached ring systems is built up from a seven-membered ring to which a six- and a three-membered ring are fused. The three six-membered rings have a twist-boat conformation, whereas the seven-membered rings display boat, chair and twist-boat conformations. The dihedral angles between the central boroxane ring and the mean planes of the attached six-membered rings are 63.67 (18), 54.89 (2) and 56.57 (19)°. The crystal packing is governed only by van der Waals inter­actions.

## Abstract 2

The title compound accounts among the rare essential oil of the Atlas cedar (*Cedrus Atlantica*) derivatives, in which three fused-ring systems surround a hexa­gonal heterocyclic ring.

## Chemical context   

Our work lies within the framework of ‘value-adding’ to the most abundant essential oils in Morocco, such as *Cedrus Atlantica*. This oil is made mainly (75%) of bicyclic sesquiterpene hydro­carbons, among which is found the compound, β-himachalene (El Haib *et al.*, 2011 ▸). The reactivity of this sesquiterpene and its derivatives has been studied extensively by our team in order to prepare new products having biological properties (El Jamili *et al.*, 2002 ▸; Zaki *et al.*, 2014 ▸; Benharref *et al.*, 2015 ▸). Indeed, these compounds were tested, using the food-poisoning technique, for their potential anti-fungal activity against the phytopathogen *Botrytis cinerea* (Daoubi *et al.*, 2004 ▸). In this work we present the crystal structure of the title compound, (1*S*,3*R*,8*R*,9*R*,10*S*)-2,4,6-tris­(2,2-di­chloro-3,7,7,10-tetra­methyl­tri­cyclo­[6.4.0.0^1,3^]dodec-9-yl)cyclo­triboroxane.

## Structural commentary   

**Figure 1:**
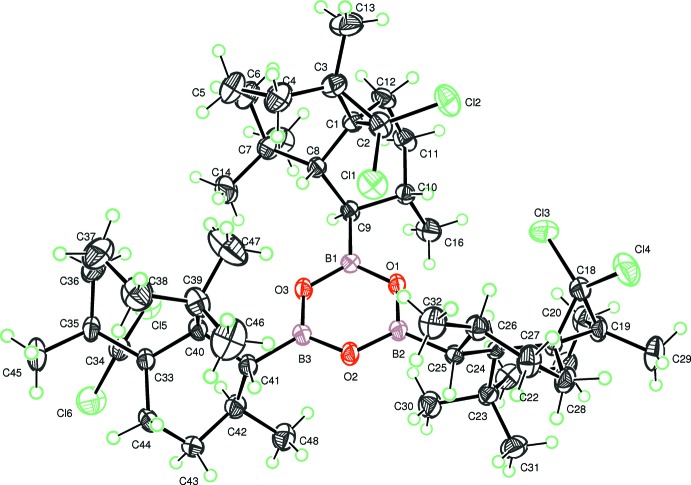
The mol­ecular structure of the title compound, showing the atom-labelling scheme. Displacement ellipsoids are drawn at the 30% probability. level. H atoms are represented as small spheres of arbitrary radii.

The mol­ecule is built up from a cyclo­triboroxane system which is linked to three identical 2,2-di­chloro- 3,7,7,10-tetra­methyl-tri­cyclo­[6.4.0.0^1^,^3^] ring systems. Each of the three attached ring systems contains a seven-membered ring, which is fused to a six-membered ring and a three-membered ring as shown in Fig. 1 ▸. The cyclo­triboroxane is virtually planar, with the largest deviation from the mean plane being 0.036 (2) Å for atom O2. The dihedral angles between the central boroxane ring and its attached six-membered rings are 63.67 (18), 54.89 (2) and 56.57 (19)°.The dihedral angles between mean planes of the six- and seven-membered rings in the three ring systems are 42.08 (19), 53.9 (2) and 67.4 (2)°. Owing to the presence of Cl atoms, the absolute configuration could be fully confirmed, by refining the Flack parameter as C1(*S*), C3(*R*), C8(*R*), C9(*R*) and C10(*S*). 
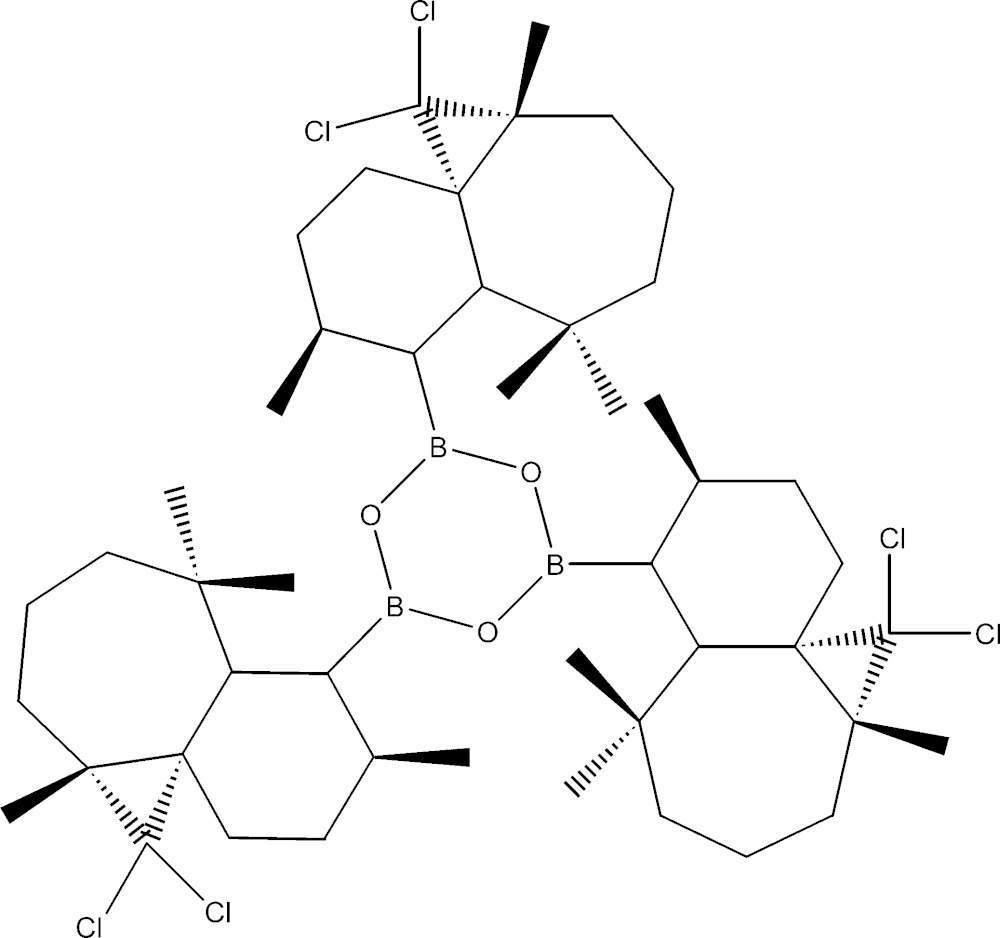


**Figure 2:**
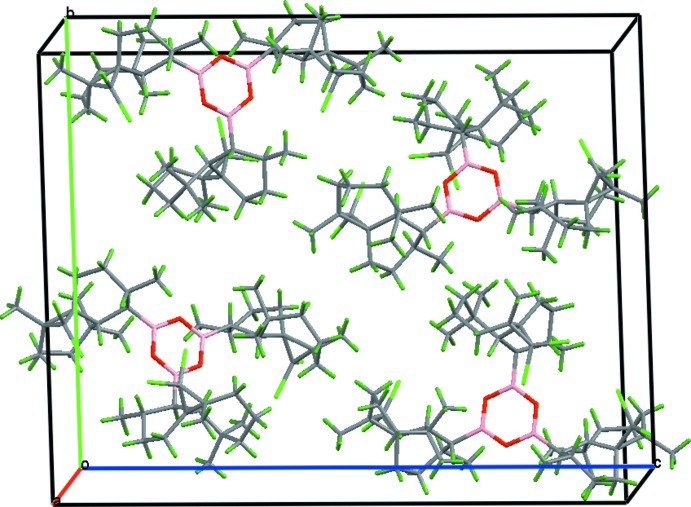
A crystal packing diagram showing mol­ecular aggregates in the unit cell.

 The crystal packing is governed only by van der Waals inter­actions (Fig. 2 ▸).

## Synthesis and crystallization   

The diborane was prepared by addition at 273 K of 2.5g (17 mmol) of boron trifluoride etherate in 0.5g (12.6 mmol) of sodium borohydride in 30 mL of diglyme. Diborane formed was driven by a stream of dry nitro­gen in 2g (7 mmol) of (1*S*,3*R*,8*R*)-2,2-di­chloro-3,7,7,10-tetra­methyl­tri­cyclo[6.4.0.0^1,3^]dodec-9-ene (El Jamili *et al.*, 2002 ▸) dissolved in 20 mL of tetra­hydro­furan at 273 K. This took about 4 h. 2 mL of sodium hydroxide (3 *N*) was then added carefully between 263 and 273 K in 15 minutes and then 2 mL of 30% hydrogen peroxide in the vicinity of 298 K. The reaction mixture was then extracted with diethyl ether, the organic phase was washed to neutrality and the solvent was evaporated under vacuum. The residue obtained was chromatographed on a column of silica gel with penta­ne–ethyl acetate (90/10), which allowed the isolation of the title compound with a yield of 35% (77 mg, 82×10^−3^ mmol). This new compound was recrystallized from ethyl acetate.

## Refinement   

Crystal data, data collection and structure refinement details are summarized in Table 1 ▸. The absolute structure was established unambiguously from anomalous dispersion effects. All H atoms were fixed geometrically and treated as riding with C—H = 0.96 Å (meth­yl), 0.97 Å (methyl­ene) and 0.98 Å (methine), and with *U*_iso_(H) = 1.2*U*_eq_(methyl­ene and methine C) or *U*_iso_(H) = 1.5*U*_eq_(methyl C).

## Supplementary Material

Crystal structure: contains datablock(s) I. DOI: 10.1107/S2056989015013390/is5406sup1.cif

Structure factors: contains datablock(s) I. DOI: 10.1107/S2056989015013390/is5406Isup2.hkl

Supporting information file. DOI: 10.1107/S2056989015013390/is5406Isup3.cml

CCDC reference: 1412287

Additional supporting information:  crystallographic information; 3D view; checkCIF report

## Figures and Tables

**Table 1:** Experimental details

Crystal data	
Chemical formula	C_48_H_75_B_3_Cl_6_O_3_
*M*_r_	945.21
Crystal system, space group	Orthorhombic, *P*2_1_2_1_2_1_
Temperature (K)	293
*a*, *b*, *c* ()	8.8240(2), 21.1340(4), 26.6620(7)
*V* (^3^)	4972.1(2)
*Z*	4
Radiation type	Mo *K*
(mm^1^)	0.39
Crystal size (mm)	0.45 0.35 0.30

Data collection	
Diffractometer	Bruker APEXII CCD
Absorption correction	Multi-scan (*SADABS*; Bruker, 2009 ▸)
*T*_min_, *T*_max_	0.642, 0.746
No. of measured, independent and observed [*I* > 2(*I*)] reflections	43691, 10143, 9010
*R*_int_	0.037
(sin /)_max_ (^1^)	0.625

Refinement	
*R*[*F* ^2^ > 2(*F* ^2^)], *wR*(*F* ^2^), *S*	0.050, 0.121, 1.15
No. of reflections	10143
No. of parameters	553
H-atom treatment	H-atom parameters constrained
_max_, _min_ (e ^3^)	0.30, 0.21
Absolute structure	Flack *x* determined using 3616 quotients [(*I* ^+^)(*I* )]/[(*I* ^+^)+(*I* )] (Parsons Flack, 2004 ▸)
Absolute structure parameter	0.021(16)

Computer programs: *APEX2* and *SAINT* (Bruker, 2009 ▸), *SHELXS2013* (Sheldrick, 2008 ▸), *SHELXL2013* (Sheldrick, 2015 ▸), *ORTEP-3 for Windows* (Farrugia, 2012 ▸) and *publCIF* (Westrip, 2010 ▸).

## supplementary crystallographic information

### Crystal data

**Table:**

C_48_H_75_B_3_Cl_6_O_3_	*D*_x_ = 1.263 Mg m^−^^3^
*M**_r_* = 945.21	Mo *K*α radiation, λ = 0.71073 Å
Orthorhombic, *P*2_1_2_1_2_1_	Cell parameters from 10143 reflections
*a* = 8.8240 (2) Å	θ = 1.5–26.4°
*b* = 21.1340 (4) Å	µ = 0.39 mm^−^^1^
*c* = 26.6620 (7) Å	*T* = 293 K
*V* = 4972.1 (2) Å^3^	Box, colourless
*Z* = 4	0.45 × 0.35 × 0.30 mm
*F*(000) = 2016	

### Data collection

**Table:**

Bruker APEXII CCD diffractometer	10143 independent reflections
Radiation source: fine-focus sealed tube	9010 reflections with *I* > 2σ(*I*)
Graphite monochromator	*R*_int_ = 0.037
ω and φ scans	θ_max_ = 26.4°, θ_min_ = 1.5°
Absorption correction: multi-scan (*SADABS*; Bruker, 2009)	*h* = −11→10
*T*_min_ = 0.642, *T*_max_ = 0.746	*k* = −26→26
43691 measured reflections	*l* = −33→26

### Refinement

**Table:**

Refinement on *F*^2^	Hydrogen site location: inferred from neighbouring sites
Least-squares matrix: full	H-atom parameters constrained
*R*[*F*^2^ > 2σ(*F*^2^)] = 0.050	*w* = 1/[σ^2^(*F*_o_^2^) + (0.0518*P*)^2^ + 1.8134*P*] where *P* = (*F*_o_^2^ + 2*F*_c_^2^)/3
*wR*(*F*^2^) = 0.121	(Δ/σ)_max_ < 0.001
*S* = 1.15	Δρ_max_ = 0.30 e Å^−^^3^
10143 reflections	Δρ_min_ = −0.21 e Å^−^^3^
553 parameters	Absolute structure: Flack *x* determined using 3616 quotients [(*I*^+^)-(*I*^-^)]/[(*I*^+^)+(*I*^-^)] (Parsons & Flack, 2004)
0 restraints	Absolute structure parameter: 0.021 (16)

### Special details

**Table:**

Geometry. All e.s.d.'s (except the e.s.d. in the dihedral angle between two l.s. planes) are estimated using the full covariance matrix. The cell e.s.d.'s are taken into account individually in the estimation of e.s.d.'s in distances, angles and torsion angles; correlations between e.s.d.'s in cell parameters are only used when they are defined by crystal symmetry. An approximate (isotropic) treatment of cell e.s.d.'s is used for estimating e.s.d.'s involving l.s. planes.

### Fractional atomic coordinates and isotropic or equivalent isotropic displacement parameters (Å^2^)

**Table:**

	*x*	*y*	*z*	*U*_iso_*/*U*_eq_	
C1	0.6458 (4)	0.17458 (16)	0.21900 (14)	0.0313 (8)	
C2	0.5394 (5)	0.22780 (19)	0.20422 (16)	0.0397 (9)	
C3	0.4910 (5)	0.1825 (2)	0.24487 (18)	0.0469 (10)	
C4	0.4921 (6)	0.2048 (3)	0.29887 (19)	0.0653 (14)	
H4A	0.3889	0.2140	0.3091	0.078*	
H4B	0.5497	0.2438	0.3010	0.078*	
C5	0.5599 (8)	0.1565 (3)	0.3353 (2)	0.0800 (18)	
H5A	0.6040	0.1793	0.3633	0.096*	
H5B	0.4782	0.1307	0.3485	0.096*	
C6	0.6803 (7)	0.1128 (3)	0.3135 (2)	0.0745 (18)	
H6A	0.6315	0.0871	0.2880	0.089*	
H6B	0.7114	0.0842	0.3401	0.089*	
C7	0.8262 (6)	0.1404 (2)	0.29000 (17)	0.0494 (11)	
C8	0.7871 (4)	0.19135 (16)	0.24919 (13)	0.0307 (7)	
H8	0.7609	0.2298	0.2679	0.037*	
C9	0.9183 (4)	0.21114 (15)	0.21272 (13)	0.0288 (7)	
H9	1.0088	0.1879	0.2234	0.035*	
C10	0.8896 (4)	0.19320 (17)	0.15719 (14)	0.0333 (8)	
H10	0.8174	0.2237	0.1432	0.040*	
C11	0.8190 (5)	0.12724 (18)	0.15311 (17)	0.0434 (10)	
H11A	0.8899	0.0963	0.1663	0.052*	
H11B	0.8022	0.1174	0.1180	0.052*	
C12	0.6687 (5)	0.12144 (18)	0.18142 (17)	0.0403 (9)	
H12A	0.5859	0.1218	0.1575	0.048*	
H12B	0.6661	0.0812	0.1989	0.048*	
C13	0.3635 (6)	0.1357 (3)	0.2337 (2)	0.0703 (16)	
H13A	0.2678	0.1547	0.2418	0.106*	
H13B	0.3774	0.0982	0.2534	0.106*	
H13C	0.3652	0.1249	0.1987	0.106*	
C14	0.9222 (7)	0.1727 (3)	0.3306 (2)	0.0724 (17)	
H14A	0.9381	0.1439	0.3579	0.109*	
H14B	0.8702	0.2097	0.3426	0.109*	
H14C	1.0182	0.1849	0.3167	0.109*	
C15	0.9206 (7)	0.0842 (2)	0.2702 (2)	0.0685 (16)	
H15A	1.0110	0.0998	0.2544	0.103*	
H15B	0.8620	0.0608	0.2463	0.103*	
H15C	0.9477	0.0571	0.2977	0.103*	
C16	1.0343 (5)	0.1964 (2)	0.12609 (18)	0.0506 (11)	
H16A	1.1059	0.1661	0.1386	0.076*	
H16B	1.0767	0.2381	0.1284	0.076*	
H16C	1.0112	0.1871	0.0917	0.076*	
C17	0.9294 (4)	0.42656 (16)	0.04345 (13)	0.0312 (8)	
C18	0.8094 (5)	0.38548 (19)	0.01951 (15)	0.0404 (9)	
C19	0.9552 (5)	0.39423 (19)	−0.00776 (14)	0.0394 (9)	
C20	1.0644 (6)	0.3389 (2)	−0.01189 (16)	0.0523 (12)	
H20A	1.0335	0.3064	0.0116	0.063*	
H20B	1.0570	0.3213	−0.0454	0.063*	
C21	1.2269 (6)	0.3558 (2)	−0.00178 (17)	0.0534 (12)	
H21A	1.2479	0.3970	−0.0162	0.064*	
H21B	1.2918	0.3252	−0.0184	0.064*	
C22	1.2659 (5)	0.3573 (2)	0.05369 (17)	0.0486 (10)	
H22A	1.3752	0.3609	0.0566	0.058*	
H22B	1.2376	0.3167	0.0679	0.058*	
C23	1.1945 (4)	0.40950 (18)	0.08649 (15)	0.0358 (8)	
C24	1.0157 (4)	0.40283 (15)	0.08946 (13)	0.0274 (7)	
H24	0.9944	0.3574	0.0920	0.033*	
C25	0.9490 (4)	0.43399 (15)	0.13811 (13)	0.0276 (7)	
H25	1.0079	0.4723	0.1450	0.033*	
C26	0.7824 (5)	0.45404 (17)	0.13025 (14)	0.0364 (8)	
H26	0.7269	0.4176	0.1169	0.044*	
C27	0.7765 (6)	0.50710 (19)	0.09091 (16)	0.0471 (10)	
H27A	0.6744	0.5099	0.0776	0.056*	
H27B	0.7996	0.5471	0.1070	0.056*	
C28	0.8875 (5)	0.49659 (17)	0.04761 (15)	0.0417 (10)	
H28A	0.8417	0.5106	0.0165	0.050*	
H28B	0.9784	0.5214	0.0532	0.050*	
C29	0.9548 (7)	0.4343 (3)	−0.05569 (16)	0.0610 (13)	
H29A	0.9150	0.4097	−0.0830	0.092*	
H29B	1.0565	0.4472	−0.0634	0.092*	
H29C	0.8927	0.4711	−0.0507	0.092*	
C30	1.2650 (5)	0.4002 (2)	0.13888 (17)	0.0521 (11)	
H30A	1.3735	0.4016	0.1363	0.078*	
H30B	1.2346	0.3599	0.1521	0.078*	
H30C	1.2309	0.4333	0.1608	0.078*	
C31	1.2463 (6)	0.4746 (2)	0.06746 (18)	0.0523 (11)	
H31A	1.2123	0.5068	0.0902	0.078*	
H31B	1.2042	0.4822	0.0348	0.078*	
H31C	1.3549	0.4755	0.0655	0.078*	
C32	0.7037 (5)	0.4753 (2)	0.17845 (17)	0.0507 (11)	
H32A	0.7632	0.5076	0.1944	0.076*	
H32B	0.6932	0.4398	0.2007	0.076*	
H32C	0.6053	0.4919	0.1705	0.076*	
C33	1.0967 (5)	0.38232 (17)	0.41633 (14)	0.0353 (9)	
C34	1.1939 (5)	0.32888 (19)	0.43607 (16)	0.0456 (10)	
C35	1.0573 (6)	0.3474 (2)	0.46558 (14)	0.0454 (10)	
C36	0.9216 (6)	0.3042 (2)	0.46380 (18)	0.0568 (12)	
H36A	0.9243	0.2762	0.4926	0.068*	
H36B	0.9275	0.2783	0.4338	0.068*	
C37	0.7722 (7)	0.3398 (3)	0.4637 (2)	0.0724 (15)	
H37A	0.7459	0.3505	0.4980	0.087*	
H37B	0.6937	0.3121	0.4508	0.087*	
C38	0.7744 (7)	0.4012 (3)	0.4321 (2)	0.0658 (14)	
H38A	0.6730	0.4189	0.4324	0.079*	
H38B	0.8405	0.4312	0.4487	0.079*	
C39	0.8254 (5)	0.3957 (2)	0.37709 (17)	0.0490 (11)	
C40	0.9966 (4)	0.37246 (17)	0.36994 (13)	0.0321 (8)	
H40	0.9923	0.3267	0.3640	0.039*	
C41	1.0726 (5)	0.40213 (17)	0.32324 (14)	0.0365 (9)	
H41	1.0393	0.4464	0.3223	0.044*	
C42	1.2457 (5)	0.40393 (19)	0.32941 (15)	0.0404 (9)	
H42	1.2800	0.3617	0.3394	0.049*	
C43	1.2839 (5)	0.45050 (19)	0.37214 (15)	0.0433 (10)	
H43A	1.3846	0.4411	0.3847	0.052*	
H43B	1.2853	0.4932	0.3588	0.052*	
C44	1.1710 (5)	0.44758 (18)	0.41581 (15)	0.0428 (10)	
H44A	1.2234	0.4549	0.4473	0.051*	
H44B	1.0944	0.4801	0.4118	0.051*	
C45	1.0765 (9)	0.3794 (3)	0.51637 (17)	0.0756 (17)	
H45A	1.0813	0.3478	0.5422	0.113*	
H45B	0.9918	0.4069	0.5225	0.113*	
H45C	1.1684	0.4037	0.5165	0.113*	
C46	0.7942 (8)	0.4625 (3)	0.3550 (2)	0.0820 (18)	
H46A	0.8149	0.4623	0.3196	0.123*	
H46B	0.8584	0.4930	0.3712	0.123*	
H46C	0.6900	0.4736	0.3605	0.123*	
C47	0.7213 (7)	0.3509 (3)	0.3493 (3)	0.089 (2)	
H47A	0.7361	0.3086	0.3616	0.134*	
H47B	0.7438	0.3523	0.3141	0.134*	
H47C	0.6179	0.3633	0.3547	0.134*	
C48	1.3317 (5)	0.4233 (2)	0.28164 (17)	0.0483 (10)	
H48A	1.3167	0.3918	0.2562	0.072*	
H48B	1.4379	0.4268	0.2890	0.072*	
H48C	1.2943	0.4633	0.2699	0.072*	
B1	0.9557 (5)	0.28394 (19)	0.21886 (16)	0.0292 (8)	
B2	0.9659 (5)	0.38880 (19)	0.18473 (15)	0.0301 (8)	
B3	1.0240 (5)	0.3724 (2)	0.27155 (16)	0.0333 (9)	
Cl1	0.58414 (13)	0.30741 (5)	0.21868 (5)	0.0522 (3)	
Cl2	0.44704 (14)	0.22716 (6)	0.14578 (5)	0.0591 (3)	
Cl3	0.75804 (16)	0.31161 (5)	0.04581 (5)	0.0609 (3)	
Cl4	0.64161 (15)	0.42021 (7)	−0.00517 (5)	0.0652 (4)	
Cl5	1.20362 (16)	0.25460 (5)	0.40531 (5)	0.0612 (3)	
Cl6	1.37512 (16)	0.34621 (7)	0.46075 (6)	0.0753 (4)	
O1	0.9416 (3)	0.32509 (11)	0.17941 (9)	0.0329 (6)	
O2	0.9998 (3)	0.41195 (11)	0.23161 (9)	0.0378 (6)	
O3	1.0025 (3)	0.30796 (12)	0.26456 (9)	0.0357 (6)	

### Atomic displacement parameters (Å^2^)

**Table:**

	*U*^11^	*U*^22^	*U*^33^	*U*^12^	*U*^13^	*U*^23^
C1	0.0350 (18)	0.0271 (16)	0.0318 (19)	−0.0024 (14)	−0.0014 (16)	0.0026 (15)
C2	0.040 (2)	0.039 (2)	0.040 (2)	0.0052 (18)	−0.0045 (18)	−0.0032 (17)
C3	0.041 (2)	0.053 (3)	0.046 (2)	−0.006 (2)	0.0063 (19)	0.004 (2)
C4	0.063 (3)	0.084 (4)	0.049 (3)	−0.010 (3)	0.020 (2)	0.001 (3)
C5	0.089 (4)	0.104 (5)	0.046 (3)	−0.026 (4)	0.015 (3)	0.015 (3)
C6	0.096 (4)	0.069 (3)	0.058 (3)	−0.025 (3)	−0.015 (3)	0.039 (3)
C7	0.063 (3)	0.038 (2)	0.047 (3)	−0.010 (2)	−0.019 (2)	0.0199 (19)
C8	0.0390 (19)	0.0246 (15)	0.0286 (18)	−0.0019 (15)	−0.0057 (16)	0.0038 (14)
C9	0.0328 (17)	0.0244 (15)	0.0292 (18)	0.0039 (14)	−0.0050 (15)	0.0013 (14)
C10	0.041 (2)	0.0279 (17)	0.0308 (19)	0.0005 (16)	−0.0015 (16)	−0.0029 (15)
C11	0.053 (2)	0.0308 (19)	0.046 (2)	0.0025 (18)	−0.006 (2)	−0.0127 (18)
C12	0.045 (2)	0.0289 (18)	0.047 (2)	−0.0047 (17)	−0.0117 (19)	−0.0057 (17)
C13	0.043 (3)	0.085 (4)	0.083 (4)	−0.021 (3)	0.002 (3)	0.014 (3)
C14	0.098 (4)	0.066 (3)	0.053 (3)	−0.021 (3)	−0.041 (3)	0.025 (3)
C15	0.078 (3)	0.035 (2)	0.093 (4)	0.003 (2)	−0.033 (3)	0.026 (2)
C16	0.049 (2)	0.054 (3)	0.049 (3)	−0.004 (2)	0.009 (2)	−0.014 (2)
C17	0.043 (2)	0.0284 (16)	0.0221 (17)	0.0018 (16)	0.0008 (16)	0.0027 (14)
C18	0.049 (2)	0.039 (2)	0.033 (2)	−0.0036 (18)	−0.0062 (18)	0.0001 (17)
C19	0.057 (3)	0.040 (2)	0.0213 (18)	−0.0003 (19)	−0.0012 (18)	−0.0020 (16)
C20	0.082 (3)	0.043 (2)	0.032 (2)	0.007 (2)	0.003 (2)	−0.0087 (18)
C21	0.069 (3)	0.049 (2)	0.042 (2)	0.014 (2)	0.022 (2)	−0.002 (2)
C22	0.044 (2)	0.048 (2)	0.054 (3)	0.009 (2)	0.010 (2)	0.007 (2)
C23	0.037 (2)	0.0383 (19)	0.033 (2)	−0.0016 (16)	0.0028 (16)	0.0042 (16)
C24	0.0388 (19)	0.0206 (15)	0.0229 (17)	−0.0011 (14)	0.0014 (15)	0.0025 (13)
C25	0.0398 (19)	0.0204 (15)	0.0227 (17)	−0.0044 (14)	−0.0011 (15)	−0.0025 (13)
C26	0.044 (2)	0.0324 (18)	0.033 (2)	0.0034 (16)	−0.0004 (18)	−0.0036 (15)
C27	0.062 (3)	0.039 (2)	0.040 (2)	0.019 (2)	−0.002 (2)	0.0024 (18)
C28	0.064 (3)	0.0281 (17)	0.033 (2)	0.0033 (18)	−0.005 (2)	0.0076 (16)
C29	0.081 (3)	0.073 (3)	0.028 (2)	0.013 (3)	0.004 (2)	0.008 (2)
C30	0.040 (2)	0.071 (3)	0.046 (3)	−0.002 (2)	−0.004 (2)	0.009 (2)
C31	0.054 (3)	0.050 (2)	0.053 (3)	−0.013 (2)	0.010 (2)	0.004 (2)
C32	0.053 (3)	0.053 (2)	0.046 (3)	0.012 (2)	0.008 (2)	−0.005 (2)
C33	0.050 (2)	0.0326 (18)	0.0231 (18)	−0.0048 (17)	−0.0034 (17)	−0.0001 (15)
C34	0.060 (3)	0.040 (2)	0.037 (2)	−0.0062 (19)	−0.013 (2)	0.0118 (18)
C35	0.070 (3)	0.045 (2)	0.0215 (19)	−0.009 (2)	−0.003 (2)	0.0054 (17)
C36	0.079 (3)	0.051 (2)	0.040 (2)	−0.012 (3)	0.010 (2)	0.009 (2)
C37	0.071 (3)	0.084 (4)	0.063 (3)	−0.012 (3)	0.021 (3)	0.007 (3)
C38	0.063 (3)	0.072 (3)	0.063 (3)	0.006 (3)	0.006 (3)	−0.011 (3)
C39	0.051 (3)	0.055 (3)	0.041 (2)	0.007 (2)	−0.004 (2)	−0.010 (2)
C40	0.043 (2)	0.0307 (17)	0.0225 (17)	−0.0037 (16)	−0.0021 (16)	−0.0042 (14)
C41	0.055 (2)	0.0300 (18)	0.0245 (18)	−0.0049 (17)	−0.0044 (18)	0.0004 (15)
C42	0.049 (2)	0.037 (2)	0.035 (2)	−0.0001 (18)	−0.0038 (18)	0.0048 (17)
C43	0.056 (3)	0.038 (2)	0.036 (2)	−0.0143 (19)	−0.008 (2)	0.0030 (17)
C44	0.066 (3)	0.034 (2)	0.028 (2)	−0.0109 (19)	−0.012 (2)	−0.0030 (16)
C45	0.118 (5)	0.084 (4)	0.025 (2)	−0.022 (4)	−0.004 (3)	0.000 (2)
C46	0.083 (4)	0.078 (4)	0.085 (4)	0.035 (3)	0.011 (4)	0.011 (3)
C47	0.054 (3)	0.117 (5)	0.098 (5)	0.006 (3)	−0.014 (3)	−0.054 (4)
C48	0.046 (2)	0.059 (3)	0.039 (2)	0.002 (2)	0.003 (2)	0.004 (2)
B1	0.031 (2)	0.0294 (18)	0.027 (2)	−0.0029 (16)	0.0009 (17)	0.0008 (16)
B2	0.039 (2)	0.0293 (19)	0.0225 (19)	−0.0029 (17)	−0.0002 (17)	−0.0015 (16)
B3	0.041 (2)	0.033 (2)	0.026 (2)	−0.0043 (18)	−0.0042 (18)	−0.0004 (17)
Cl1	0.0580 (6)	0.0354 (5)	0.0632 (7)	0.0129 (5)	−0.0028 (6)	−0.0066 (5)
Cl2	0.0518 (6)	0.0700 (7)	0.0555 (7)	0.0085 (6)	−0.0222 (6)	0.0032 (6)
Cl3	0.0764 (8)	0.0435 (5)	0.0629 (7)	−0.0235 (6)	−0.0024 (6)	−0.0012 (5)
Cl4	0.0557 (7)	0.0844 (9)	0.0557 (7)	0.0024 (6)	−0.0206 (6)	0.0005 (7)
Cl5	0.0801 (9)	0.0399 (5)	0.0637 (7)	0.0109 (6)	−0.0055 (7)	0.0077 (5)
Cl6	0.0662 (8)	0.0823 (9)	0.0772 (9)	−0.0134 (7)	−0.0370 (7)	0.0323 (8)
O1	0.0496 (15)	0.0270 (12)	0.0220 (12)	−0.0050 (11)	−0.0058 (12)	0.0006 (10)
O2	0.0651 (18)	0.0259 (12)	0.0226 (13)	−0.0054 (12)	−0.0041 (12)	−0.0008 (10)
O3	0.0556 (16)	0.0276 (12)	0.0240 (13)	−0.0053 (12)	−0.0076 (12)	0.0035 (10)

### Geometric parameters (Å, º)

**Table:**

C1—C2	1.517 (5)	C26—C32	1.529 (6)
C1—C12	1.519 (5)	C26—C27	1.536 (6)
C1—C8	1.526 (5)	C26—H26	0.9800
C1—C3	1.540 (6)	C27—C28	1.531 (6)
C2—C3	1.508 (6)	C27—H27A	0.9700
C2—Cl2	1.758 (4)	C27—H27B	0.9700
C2—Cl1	1.771 (4)	C28—H28A	0.9700
C3—C4	1.515 (7)	C28—H28B	0.9700
C3—C13	1.527 (7)	C29—H29A	0.9600
C4—C5	1.531 (8)	C29—H29B	0.9600
C4—H4A	0.9700	C29—H29C	0.9600
C4—H4B	0.9700	C30—H30A	0.9600
C5—C6	1.523 (9)	C30—H30B	0.9600
C5—H5A	0.9700	C30—H30C	0.9600
C5—H5B	0.9700	C31—H31A	0.9600
C6—C7	1.547 (7)	C31—H31B	0.9600
C6—H6A	0.9700	C31—H31C	0.9600
C6—H6B	0.9700	C32—H32A	0.9600
C7—C14	1.535 (7)	C32—H32B	0.9600
C7—C15	1.544 (7)	C32—H32C	0.9600
C7—C8	1.569 (5)	C33—C34	1.513 (6)
C8—C9	1.569 (5)	C33—C44	1.527 (5)
C8—H8	0.9800	C33—C40	1.534 (5)
C9—C10	1.549 (5)	C33—C35	1.545 (5)
C9—B1	1.582 (5)	C34—C35	1.492 (7)
C9—H9	0.9800	C34—Cl6	1.768 (5)
C10—C16	1.524 (6)	C34—Cl5	1.773 (4)
C10—C11	1.530 (5)	C35—C36	1.507 (7)
C10—H10	0.9800	C35—C45	1.523 (6)
C11—C12	1.532 (6)	C36—C37	1.518 (8)
C11—H11A	0.9700	C36—H36A	0.9700
C11—H11B	0.9700	C36—H36B	0.9700
C12—H12A	0.9700	C37—C38	1.547 (8)
C12—H12B	0.9700	C37—H37A	0.9700
C13—H13A	0.9600	C37—H37B	0.9700
C13—H13B	0.9600	C38—C39	1.538 (7)
C13—H13C	0.9600	C38—H38A	0.9700
C14—H14A	0.9600	C38—H38B	0.9700
C14—H14B	0.9600	C39—C47	1.513 (7)
C14—H14C	0.9600	C39—C46	1.554 (7)
C15—H15A	0.9600	C39—C40	1.600 (6)
C15—H15B	0.9600	C40—C41	1.547 (5)
C15—H15C	0.9600	C40—H40	0.9800
C16—H16A	0.9600	C41—C42	1.536 (6)
C16—H16B	0.9600	C41—B3	1.575 (5)
C16—H16C	0.9600	C41—H41	0.9800
C17—C18	1.511 (5)	C42—C48	1.538 (6)
C17—C24	1.529 (5)	C42—C43	1.543 (6)
C17—C28	1.529 (5)	C42—H42	0.9800
C17—C19	1.544 (5)	C43—C44	1.533 (6)
C18—C19	1.489 (6)	C43—H43A	0.9700
C18—Cl3	1.770 (4)	C43—H43B	0.9700
C18—Cl4	1.779 (4)	C44—H44A	0.9700
C19—C20	1.518 (6)	C44—H44B	0.9700
C19—C29	1.533 (6)	C45—H45A	0.9600
C20—C21	1.502 (7)	C45—H45B	0.9600
C20—H20A	0.9700	C45—H45C	0.9600
C20—H20B	0.9700	C46—H46A	0.9600
C21—C22	1.519 (7)	C46—H46B	0.9600
C21—H21A	0.9700	C46—H46C	0.9600
C21—H21B	0.9700	C47—H47A	0.9600
C22—C23	1.543 (6)	C47—H47B	0.9600
C22—H22A	0.9700	C47—H47C	0.9600
C22—H22B	0.9700	C48—H48A	0.9600
C23—C31	1.537 (6)	C48—H48B	0.9600
C23—C30	1.542 (6)	C48—H48C	0.9600
C23—C24	1.586 (5)	B1—O1	1.370 (5)
C24—C25	1.569 (5)	B1—O3	1.383 (5)
C24—H24	0.9800	B2—O1	1.371 (5)
C25—C26	1.544 (5)	B2—O2	1.375 (5)
C25—B2	1.574 (5)	B3—O2	1.371 (5)
C25—H25	0.9800	B3—O3	1.387 (5)
			
C2—C1—C12	117.3 (3)	C32—C26—C27	110.1 (3)
C2—C1—C8	118.1 (3)	C32—C26—C25	113.5 (3)
C12—C1—C8	114.3 (3)	C27—C26—C25	109.0 (3)
C2—C1—C3	59.1 (3)	C32—C26—H26	108.0
C12—C1—C3	119.6 (3)	C27—C26—H26	108.0
C8—C1—C3	117.6 (3)	C25—C26—H26	108.0
C3—C2—C1	61.2 (3)	C28—C27—C26	112.8 (3)
C3—C2—Cl2	120.0 (3)	C28—C27—H27A	109.0
C1—C2—Cl2	120.7 (3)	C26—C27—H27A	109.0
C3—C2—Cl1	120.6 (3)	C28—C27—H27B	109.0
C1—C2—Cl1	120.7 (3)	C26—C27—H27B	109.0
Cl2—C2—Cl1	107.7 (2)	H27A—C27—H27B	107.8
C2—C3—C4	119.0 (4)	C17—C28—C27	110.5 (3)
C2—C3—C13	118.6 (4)	C17—C28—H28A	109.6
C4—C3—C13	113.1 (4)	C27—C28—H28A	109.6
C2—C3—C1	59.7 (3)	C17—C28—H28B	109.6
C4—C3—C1	117.0 (4)	C27—C28—H28B	109.6
C13—C3—C1	119.7 (4)	H28A—C28—H28B	108.1
C3—C4—C5	113.5 (5)	C19—C29—H29A	109.5
C3—C4—H4A	108.9	C19—C29—H29B	109.5
C5—C4—H4A	108.9	H29A—C29—H29B	109.5
C3—C4—H4B	108.9	C19—C29—H29C	109.5
C5—C4—H4B	108.9	H29A—C29—H29C	109.5
H4A—C4—H4B	107.7	H29B—C29—H29C	109.5
C6—C5—C4	115.8 (4)	C23—C30—H30A	109.5
C6—C5—H5A	108.3	C23—C30—H30B	109.5
C4—C5—H5A	108.3	H30A—C30—H30B	109.5
C6—C5—H5B	108.3	C23—C30—H30C	109.5
C4—C5—H5B	108.3	H30A—C30—H30C	109.5
H5A—C5—H5B	107.4	H30B—C30—H30C	109.5
C5—C6—C7	120.4 (5)	C23—C31—H31A	109.5
C5—C6—H6A	107.2	C23—C31—H31B	109.5
C7—C6—H6A	107.2	H31A—C31—H31B	109.5
C5—C6—H6B	107.2	C23—C31—H31C	109.5
C7—C6—H6B	107.2	H31A—C31—H31C	109.5
H6A—C6—H6B	106.9	H31B—C31—H31C	109.5
C14—C7—C15	106.6 (4)	C26—C32—H32A	109.5
C14—C7—C6	109.9 (5)	C26—C32—H32B	109.5
C15—C7—C6	107.3 (4)	H32A—C32—H32B	109.5
C14—C7—C8	107.8 (3)	C26—C32—H32C	109.5
C15—C7—C8	114.2 (4)	H32A—C32—H32C	109.5
C6—C7—C8	110.9 (4)	H32B—C32—H32C	109.5
C1—C8—C9	109.8 (3)	C34—C33—C44	115.7 (4)
C1—C8—C7	112.7 (3)	C34—C33—C40	120.4 (3)
C9—C8—C7	116.8 (3)	C44—C33—C40	111.3 (3)
C1—C8—H8	105.5	C34—C33—C35	58.4 (3)
C9—C8—H8	105.5	C44—C33—C35	122.3 (3)
C7—C8—H8	105.5	C40—C33—C35	119.4 (3)
C10—C9—C8	114.0 (3)	C35—C34—C33	61.9 (3)
C10—C9—B1	111.8 (3)	C35—C34—Cl6	118.7 (3)
C8—C9—B1	110.4 (3)	C33—C34—Cl6	119.2 (3)
C10—C9—H9	106.7	C35—C34—Cl5	121.1 (3)
C8—C9—H9	106.7	C33—C34—Cl5	121.8 (3)
B1—C9—H9	106.7	Cl6—C34—Cl5	108.2 (3)
C16—C10—C11	110.1 (3)	C34—C35—C36	117.8 (4)
C16—C10—C9	111.8 (3)	C34—C35—C45	119.7 (5)
C11—C10—C9	111.0 (3)	C36—C35—C45	112.7 (4)
C16—C10—H10	108.0	C34—C35—C33	59.7 (3)
C11—C10—H10	108.0	C36—C35—C33	116.2 (3)
C9—C10—H10	108.0	C45—C35—C33	121.3 (4)
C10—C11—C12	113.0 (3)	C35—C36—C37	112.9 (4)
C10—C11—H11A	109.0	C35—C36—H36A	109.0
C12—C11—H11A	109.0	C37—C36—H36A	109.0
C10—C11—H11B	109.0	C35—C36—H36B	109.0
C12—C11—H11B	109.0	C37—C36—H36B	109.0
H11A—C11—H11B	107.8	H36A—C36—H36B	107.8
C1—C12—C11	112.4 (3)	C36—C37—C38	113.9 (4)
C1—C12—H12A	109.1	C36—C37—H37A	108.8
C11—C12—H12A	109.1	C38—C37—H37A	108.8
C1—C12—H12B	109.1	C36—C37—H37B	108.8
C11—C12—H12B	109.1	C38—C37—H37B	108.8
H12A—C12—H12B	107.9	H37A—C37—H37B	107.7
C3—C13—H13A	109.5	C39—C38—C37	117.4 (5)
C3—C13—H13B	109.5	C39—C38—H38A	107.9
H13A—C13—H13B	109.5	C37—C38—H38A	107.9
C3—C13—H13C	109.5	C39—C38—H38B	107.9
H13A—C13—H13C	109.5	C37—C38—H38B	107.9
H13B—C13—H13C	109.5	H38A—C38—H38B	107.2
C7—C14—H14A	109.5	C47—C39—C38	109.6 (5)
C7—C14—H14B	109.5	C47—C39—C46	106.0 (5)
H14A—C14—H14B	109.5	C38—C39—C46	104.0 (4)
C7—C14—H14C	109.5	C47—C39—C40	108.8 (4)
H14A—C14—H14C	109.5	C38—C39—C40	114.4 (4)
H14B—C14—H14C	109.5	C46—C39—C40	113.7 (4)
C7—C15—H15A	109.5	C33—C40—C41	110.1 (3)
C7—C15—H15B	109.5	C33—C40—C39	113.9 (3)
H15A—C15—H15B	109.5	C41—C40—C39	112.4 (3)
C7—C15—H15C	109.5	C33—C40—H40	106.6
H15A—C15—H15C	109.5	C41—C40—H40	106.6
H15B—C15—H15C	109.5	C39—C40—H40	106.6
C10—C16—H16A	109.5	C42—C41—C40	110.8 (3)
C10—C16—H16B	109.5	C42—C41—B3	112.0 (3)
H16A—C16—H16B	109.5	C40—C41—B3	115.1 (3)
C10—C16—H16C	109.5	C42—C41—H41	106.1
H16A—C16—H16C	109.5	C40—C41—H41	106.1
H16B—C16—H16C	109.5	B3—C41—H41	106.1
C18—C17—C24	120.0 (3)	C41—C42—C48	114.1 (3)
C18—C17—C28	114.7 (3)	C41—C42—C43	108.2 (3)
C24—C17—C28	112.3 (3)	C48—C42—C43	109.5 (3)
C18—C17—C19	58.4 (3)	C41—C42—H42	108.3
C24—C17—C19	119.4 (3)	C48—C42—H42	108.3
C28—C17—C19	121.9 (3)	C43—C42—H42	108.3
C19—C18—C17	61.9 (3)	C44—C43—C42	113.1 (3)
C19—C18—Cl3	121.6 (3)	C44—C43—H43A	109.0
C17—C18—Cl3	121.3 (3)	C42—C43—H43A	109.0
C19—C18—Cl4	119.2 (3)	C44—C43—H43B	109.0
C17—C18—Cl4	120.2 (3)	C42—C43—H43B	109.0
Cl3—C18—Cl4	107.3 (2)	H43A—C43—H43B	107.8
C18—C19—C20	119.2 (4)	C33—C44—C43	108.8 (3)
C18—C19—C29	118.3 (4)	C33—C44—H44A	109.9
C20—C19—C29	111.5 (4)	C43—C44—H44A	109.9
C18—C19—C17	59.7 (3)	C33—C44—H44B	109.9
C20—C19—C17	119.9 (3)	C43—C44—H44B	109.9
C29—C19—C17	119.5 (3)	H44A—C44—H44B	108.3
C21—C20—C19	114.2 (4)	C35—C45—H45A	109.5
C21—C20—H20A	108.7	C35—C45—H45B	109.5
C19—C20—H20A	108.7	H45A—C45—H45B	109.5
C21—C20—H20B	108.7	C35—C45—H45C	109.5
C19—C20—H20B	108.7	H45A—C45—H45C	109.5
H20A—C20—H20B	107.6	H45B—C45—H45C	109.5
C20—C21—C22	113.3 (4)	C39—C46—H46A	109.5
C20—C21—H21A	108.9	C39—C46—H46B	109.5
C22—C21—H21A	108.9	H46A—C46—H46B	109.5
C20—C21—H21B	108.9	C39—C46—H46C	109.5
C22—C21—H21B	108.9	H46A—C46—H46C	109.5
H21A—C21—H21B	107.7	H46B—C46—H46C	109.5
C21—C22—C23	118.3 (4)	C39—C47—H47A	109.5
C21—C22—H22A	107.7	C39—C47—H47B	109.5
C23—C22—H22A	107.7	H47A—C47—H47B	109.5
C21—C22—H22B	107.7	C39—C47—H47C	109.5
C23—C22—H22B	107.7	H47A—C47—H47C	109.5
H22A—C22—H22B	107.1	H47B—C47—H47C	109.5
C31—C23—C30	107.0 (4)	C42—C48—H48A	109.5
C31—C23—C22	109.4 (3)	C42—C48—H48B	109.5
C30—C23—C22	104.9 (3)	H48A—C48—H48B	109.5
C31—C23—C24	113.1 (3)	C42—C48—H48C	109.5
C30—C23—C24	110.2 (3)	H48A—C48—H48C	109.5
C22—C23—C24	111.8 (3)	H48B—C48—H48C	109.5
C17—C24—C25	109.8 (3)	O1—B1—O3	118.1 (3)
C17—C24—C23	115.2 (3)	O1—B1—C9	121.2 (3)
C25—C24—C23	112.1 (3)	O3—B1—C9	120.7 (3)
C17—C24—H24	106.3	O1—B2—O2	118.5 (3)
C25—C24—H24	106.3	O1—B2—C25	120.0 (3)
C23—C24—H24	106.3	O2—B2—C25	121.5 (3)
C26—C25—C24	111.1 (3)	O2—B3—O3	118.2 (3)
C26—C25—B2	111.3 (3)	O2—B3—C41	118.6 (3)
C24—C25—B2	111.3 (3)	O3—B3—C41	123.2 (3)
C26—C25—H25	107.7	B1—O1—B2	122.0 (3)
C24—C25—H25	107.7	B3—O2—B2	121.5 (3)
B2—C25—H25	107.7	B1—O3—B3	121.3 (3)
